# Psychological Outcomes on Anxiety and Depression after Interventions for Temporomandibular Disorders: A Systematic Review and Meta-Analysis

**DOI:** 10.3390/diagnostics13040653

**Published:** 2023-02-09

**Authors:** Lai Ying Luo, Johyun Lee, Kar Yan Li, Yiu Yan Leung, Dion Tik Shun Li

**Affiliations:** 1 Oral and Maxillofacial Surgery, Faculty of Dentistry, The University of Hong Kong, Hong Kong SAR, China; 2 Faculty of Dentistry, The University of Hong Kong, Hong Kong SAR, China; 3 Clinical Research Centre, Faculty of Dentistry, The University of Hong Kong, Hong Kong SAR, China

**Keywords:** temporomandibular joint disorders, depression, anxiety disorders, psychological distress

## Abstract

Many studies have shown mutual interaction between temporomandibular disorders (TMD) and psychological distress. However, evidence on the effectiveness of therapeutic interventions for TMD on psychological outcomes is scarce. This review aimed to summarise the best evidence on the association between interventions for TMD and psychological outcomes regarding symptoms of anxiety and depression. Electronic search was carried out in databases, including Pubmed, Web of Science, Medline, Cochrane Library, and Scopus. All eligible studies were included for narrative synthesis. Eligible randomised controlled trials (RCTs) were included for the meta-analysis. The overall effect size of interventions for TMD was analysed in standardised mean difference (SMD) in levels of anxiety and depression. Ten studies were included in the systematic review. Of these, nine were included in the narrative analysis and four were included in the meta-analysis. All included studies and the result of the narrative analysis showed a statistically significant beneficial effect of interventions for TMD on improving symptoms of anxiety and depression (*p* < 0.0001); however, a statistically significant overall effect was not found in the meta-analyses. Current evidence is in favour of the interventions for TMD in improving symptoms of depression and anxiety. However, the effect is statistically uncertain and warrants future studies to enable the best synthesis of the evidence.

## 1. Introduction

Temporomandibular disorders (TMD) are commonly defined as a group of orofacial disorders involving the masticatory muscles, the temporomandibular joint (TMJ), and adjacent structures with traumatic, neoplastic, and/or musculoskeletal disorders as aetiology [1,2]. Patients often present with a wide and complex range of clinical conditions, including painful conditions, such as myalgia, arthralgia, referred pain, and headache attributed to TMD, and non-painful conditions, such as disc displacement, limited opening, degenerative joint disease, and subluxation [3].

TMD affects 5–15% of adults in general, as reported in different studies, while TMD-related symptoms have been reported to be up to 50% of adults [4]. A recent systematic review and meta-analysis has reported the overall prevalence of TMD diagnosed by the research diagnostic criteria (RDC/TMD) or diagnostic criteria (DC/TMD) to be approximately 31% for adults and elderly [5].

TMD is a common orofacial pain disease, which affects a significant percentage of the population, yet its diagnosis and management remain a challenge. There is a lack of consensus in many aspects because of its multifactorial aetiologies. Although the aetiology of TMD is complex and still not clearly understood, it is generally believed to comprise of biological, psychological, and social factors [6,7]. Therefore, it is important to also consider the psychological symptoms during diagnosis of the disease. The design of DC/TMD and RDC/TMD, the two most widely accepted and standardised assessment tools, has validated the importance of psychological assessment by including psychosocial (Axis II) diagnoses in the dual-axis biopsychosocial diagnostic tool [3].

Depressive and anxiety disorders are the two most common mental disorders, affecting 280 and 301 million people around the world, respectively [8]. Depression is characterised by depressed mood, loss of pleasure or interest in activities, poor concentration, low self-worth, disrupted sleep, change of appetite or weight, and low energy. Patients suffering from depression have a higher risk of committing suicide. Anxiety disorder is characterised by excessive fear, panic attacks, worry in social situations, sleep disturbance, fatigue, sense of tension, nervousness, and restlessness [9,10].

Over the decades, many studies have demonstrated positive correlations between TMD and symptoms of anxiety and depression. In the systematic review of De La Torre Canales et al., a high prevalence of moderate–to–severe depression was observed to range from 21.4 to 60.1% in patients diagnosed with TMD [11]. According to Florjański et al.’s recent literature review, despite the correlation between anxiety and TMD being more controversial when compared to that of depression, the higher prevalence of trait-anxiety (one subtype of anxiety) among patients with TMD than healthy individuals was consistent [9].

The role of a dental surgeon is to detect any symptoms of depression and/or anxiety in patients diagnosed with TMD rather than to diagnose a mental disorder. The most used screening tools, such as the Beck Depression and Anxiety Inventories and the Symptom Checklist-90-Reivsed (SCL-90), are generally questionnaires to reflect patients’ self-reported measures.

TMD being a significant and complex health issue, debates are not only over the aetiologies, but also its management. Treatment options range from conservative measures, such as analgesics, occlusal splints, and physiotherapy, to minimally invasive options, such as arthroscopy, arthrocentesis, and intra-articular injection, to open joint surgery. There are also non-standard treatment options, such as Botox injection, acupuncture, and extracorporeal shockwave therapy [4].

Despite the wide variety of options for intervention, there is an increasing consensus on using a multimodal approach in the management of TMD. More studies have supported the concept of the more comprehensive biopsychosocial model of aetiology instead of the more narrowly focused historical biomedical model, especially for providing an integrated and hence successful management of the disease [12]. It is emphasised to manage TMD as a multidimensional chronic illness by a rehabilitation approach that allows integrated assessment between physical and psychological symptoms, and to treat not only the “disorder”, but also the “illness” [13]. Therefore, it is essential to evaluate the efficacy of therapeutic intervention for TMD towards not just the primary treatment outcome, but also the secondary psychological outcomes.

In the literature regarding TMD, most studies have investigated the prevalence and aetiology of the disease. There has been increasing evidence of concurrence and mutual interaction between TMD and anxiety and depression [14]. It is reasonable to suggest that a successful intervention for TMD might improve patients’ depression and anxiety symptoms. However, we found no reviews that evaluate the influence of treatments of TMD towards the psychological conditions of the patients.

The objective of this systematic review and meta-analysis was to summarise the best evidence on the association between psychological status (i.e., anxiety and depression) and the outcome of therapeutic interventions for TMD.

## 2. Materials and Methods

This systematic review was conducted following the Preferred Reporting Items for Systematic Reviews and Meta-Analyses (PRISMA) guidelines. The research protocol was registered with the International Prospective Register of Systematic Reviews (PROSPERO), number CRD42022324116 †‡ († The protocol was registered and published during the period of COVID-19 pandemic. Submissions which passed a basic automated check were published automatically after 30 days of waiting time, in order to allow the PROSPERO team to focus on COVID-19 related reviews. Eligibility of this protocol was not checked by the PROSPERO team before this study was commenced. ‡ The registered protocol was amended to also include studies without control groups in order to increase the variety of studies to review).

### 2.1. Study Selection

Population

Studies reporting adult patients diagnosed with TMD using the RDC/TMD (Axis I and/or Axis II) or its revisions or the new DC/TMD instruments were included. Studies of patients diagnosed with pain disorders other than TMD were excluded.

Intervention

All standard treatment options for TMD identified with the goal to improve the disease by reducing pain and/or improving jaw function were included if they were systematically delivered to the subjects according to a pre-defined algorithm or protocol and were started and completed during the perioperative period of the studies. These included conservative options, including medications (such as analgesics, non-steroidal anti-inflammatory drugs, and muscle relaxants), occlusal appliances of various designs, physiotherapy (such as muscle training and massage), changing of behaviour (soft diet and rest), minimally invasive options (such as arthroscopy, arthrocentesis and intra-articular injections, and open joint surgical options (such as disc repositioning procedures, removal of osteophytes, removal of pathologic tissue, biopsy of the TMJ and alloplastic replacement of the TMJ). Botox injection, acupuncture, extracorporeal shock wave therapy, and laser auriculotherapy, which are currently not considered standard treatment options of TMD, were excluded. Psychological interventions, such as anti-depressants, counselling, stress coping strategies, etc., were not defined as interventions for TMD in this review.

Controls

Studies that have reported comparative groups of subjects receiving no treatments, placebo treatments, or interventions other than the standard treatment options for TMD mentioned above were categorised as studies with control groups. These comparative groups were analysed under the same subgroup in the meta-analysis.

Outcome

Studies included had to report on psychological outcome regarding the severity of anxiety or/and depression. Assessment tools of anxiety included the State-Trait Anxiety Inventory (STAI), Beck Anxiety Inventory (BAI), Hospital Anxiety and Depression Scale (HADS), General Anxiety Disorder-7 (GAD-7) and SCL-90, while those of depression included the Beck Depression Inventory (BDI), Center for Epidemiologic Studies Depression Scale (CES-D), Geriatric Depression Scale (GDS), Patient Health Questionnaire-9 (PHQ-9), HADS and SCL-90.

### 2.2. Summary of Eligible Criteria

Inclusion criteria

1. Studies conducted among subjects diagnosed with TMD

2. At least one intervention for TMD was delivered

3. Studies reporting outcomes on depression/anxiety after TMD interventions

4. Studies in the English language

Exclusion criteria

1. Studies in animals

2. Studies conducted in children/adolescents aged below 18

3. Studies conducted in patients with other pain disorders, except TMD

4. Studies not using DC/TMD or RDC/TMD for definitive diagnosis

5. Articles with incomplete information

6. Systematic reviews/meta-analyses, meeting/congress reports, and retrospective studies

### 2.3. Search Strategy

Electronic search was carried out in databases, including Pubmed, Web of Science, Medline, Cochrane Library, and Scopus. The literature search was constructed around search terms for “TMD”, “depression”, and “anxiety” (Table 1). No restrictions were considered regarding publication year or language. Titles and/or abstracts were reviewed after the elimination of duplicates to exclude seemingly irrelevant articles. Manual search was then performed through the bibliographical references of these articles. These potentially relevant articles were further screened by applying the inclusion and exclusion criteria mentioned above by two independent reviewers. A third independent reviewer (a senior researcher) was consulted on any cases of persisting disagreement. The total search of all databases was performed within March 2022.

### 2.4. Data Management

The full texts of the articles included were retrieved. Detailed data were extracted from articles independently by two authors according to the data collection form, including information on the author, year of publication, country of publication, study design, size of the population at baseline, characteristics of the population (age at baseline, distribution of experimental, and control groups), duration of follow-up, diagnostic tools of TMD, types of interventions for TMD, outcome measure of TMD intervention, assessment tools of anxiety or/and depression, number of subjects included in the analysis (number of subjects in total, experimental, and control groups), change in treatment outcome of TMD, and severity of anxiety or/and depression before and after interventions.

### 2.5. Assessment of Risk of Bias and Quality Evaluation

Risks of bias were independently rated by two reviewers based on version 2 of the Cochrane risk-of-bias tool for randomised trials (RoB 2) for randomised controlled studies, based on five domains: bias arising from the randomization process; bias due to deviations from intended interventions; bias due to missing outcome data; bias in measurement of the outcome; and bias in selection of the reported result. A risk-of-bias judgement was reached for each domain, then an overall judgment, by assigning one of the three levels: low risk of bias; some concerns; or high risk of bias [15].

A modified Newcastle–Ottawa Quality Assessment Scale was designed to evaluate the quality of all studies included in this review, with reference to the original assessment scale for cohort studies [16]. A “star system” was employed to judge each study based on three main domains: the selection of the sample, the comparability of the groups, and the ascertainment of the outcome. A maximum of three stars for “Selection”, one star for “Comparability”, and three stars for “Outcome”, which made up a maximum of seven stars that could be scored by each study. This modified questionnaire was designed to provide a quick and direct critical appraisal of the included studies. A study with a total score of 6–7 was categorised as good quality, 3–5 as fair quality, and 0–2 as poor quality. The detailed questionnaire is available in Appendix A.

A third independent reviewer (a senior researcher) was consulted on any discrepancies until consensus was reached.

### 2.6. Data Analysis

The meta-analyses were performed using the Review Manager (RevMan) 5 software (Version 5.4, The Nordic Cochrane Centre, Copenhagen) when at least two studies reporting specific outcomes were available. A fixed effects model was employed because only a small number of studies (i.e., less than five) were eligible to be included in each analysis [17,18]. All *p*-values were reported, and *p* ≤ 0.05 was considered as being statistically significant.

### 2.7. Meta-Analyses including Only Studies with Control Groups

The effects of interventions for TMD on depression and anxiety, compared to control interventions, were analysed.

Standardised mean difference (SMD) was used as a summary statistic in the meta-analysis since all studies assessed the same outcome, but with various measurement tools (for example, Costa et al. [19] used HADS, while Alajbeg et al. [20] used PHQ-9 in measuring the degree of depression). A SMD allowed standardization of the results of various studies to a uniform scale for analysis. It is calculated as the difference in mean outcomes between the intervention and control groups, divided by the standard deviation (SD) of the outcome among participants, with 95% confidence intervals (CIs) [15]. When the SDs were unavailable, they were estimated by calculation, using standard errors, Cls, t-values, interquartile deviations, and/or the correlation coefficient [15,21]. The correlation coefficient was obtained from calculation using reported data in Alajbeg et al.’s study [18], which was reported in considerable detail. The mean differences, when not reported, were calculated by subtracting the post-intervention measurement from the baseline measurement. Measurements taking the closest to the beginning and the end of the interventions were chosen for calculation when more than one baseline and/or post-intervention measurement was reported.

A positive SMD was defined to represent the beneficial effects of interventions for TMD compared to the control intervention for all outcomes (e.g., improvement in the levels of pain, depression, and/or anxiety). A combined SMD was computed in RevMan when there were more than one intervention group (for example, in Melo et al.’s study [22], there were three intervention groups: occlusal splint, manual therapy, and combined therapy) using the mean difference and SD of each group [15]. Improvement was defined as reduction in the levels of pain, depression, and/or anxiety in all statistical analyses in this review.

The overall effect size was evaluated by interpreting the SMDs using the Cohen’s categories, where SMD = 0.2 to 0.5 represents a small effect, SMD = 0.5 to 0.8 a moderate effect, and SMD > 0.8 a large effect [23].

The certainty of the evidence of each outcome was evaluated by the Grades of Recommendation, Assessment, Development, and Evaluation (GRADE) approach by two independent reviewers. Five GRADE considerations were used for assessment, including risk of bias, consistency of effect, imprecision, indirectness, and publication bias [15,24].

### 2.8. Assessment of Heterogeneity

The statistical heterogeneity was assessed by a chi-squared (χ^2^) test and inconsistency (I^2^) statistics. A rough guide to interpret I^2^ was as follows: 0 to 40%: might not be important; 30 to 60%: moderate; 50 to 90%: substantial; and 70 to 100%: considerable. Considering the low power of the χ^2^ test when only a few studies were included in an analysis, a *p*-value of ≤0.10 was used to indicate significant heterogeneity.

### 2.9. Narrative Analysis including All Studies

Narrative syntheses of the mean difference between the outcomes before and after interventions in all studies (including those without control groups) were conducted by obtaining the mean change and standard error (SE) in each intervention group. When the SEs were unavailable, they were estimated using the SDs and the sample size of the groups [15]. The findings were interpreted with caution because any placebo effect or effects due to background inclusion were not excluded in these analyses. Neither judgement of the overall effect size nor the certainty of evidence was derived to eliminate possible misinterpretations.

## 3. Results

### 3.1. Literature Search

**Figure 1:**
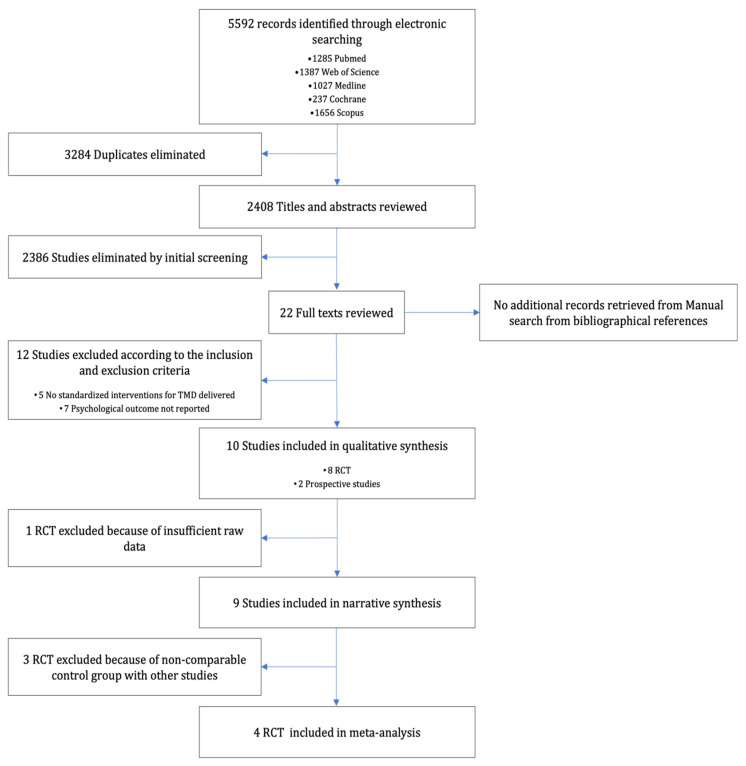
PRISMA flowchart of the result of literature search.

5592 records were retrieved through the electronic search, and 2408 records were screened after the elimination of duplicates. After the review of titles and/or abstracts, 2386 irrelevant records were excluded because their diagnoses for TMD were not by DC/TMD or RDC/TMD or their variations, and/or there were no interventions for TMD carried out. Out of the 22 full texts reviewed, 12 of them were excluded after being assessed for eligibility because either psychological outcomes were not reported [25,26,27,28,29,30,31], or no standard interventions for TMD were delivered [32,33,34,35,36]. No additional records were retrieved after manual search through the reference lists of identified articles. Among the 10 studies (8 RCTs and 2 non-randomised clinical trials) included for qualitative review, 1 RCT [37] was excluded from any quantitative analyses because of insufficient statistical details. A total of 9 studies with 713 patients were included in the narrative analysis. Three RCTs were further excluded from the meta-analysis because either all subjects received interventions for TMD, including the control group (i.e., conservative treatments for TMD) [38], or the assigned interventions were not considered to be standard treatment options [39,40]. Finally, 4 RCTs with 203 patients were eligible and included in the meta-analysis (Figure 1).

### 3.2. Study Characteristics

Population characteristics

The summary characteristics of the included studies are presented in Table 2. Of the 10 studies, 5 originated from South America (Brazil) [19,22,37,39,40]; 3 from Europe (Croatia [20], Romania [41], and Portugal [42]); and 2 from North America (USA) [38,43]. The majority of the sample population were made up of patients recruited from dental school clinics [20,22,37,40,41,42,43], and the others from private dental clinics [41] and recruitment among local community [19] and primary school teachers [39]. One study only recruited elderly aged 60–79 years [41], while one only recruited female patients [40].

A total of 736 subjects were included in this review. All patients were diagnosed with TMD using RDC/TMD [19,22,37,38,39,40,41,43] or DC/TMD [20,42]. In total, 20% of them were specifically diagnosed with myofascial pain or myalgia [19,39,40,42]. Females made up most of the sample population in all included studies, ranging between 69.1 and 100% (median: 87.9%). The mean age ranged from 25.9 to 68.72 years, with a median age of 36 years. Race was only reported in three studies [20,37,38].

Intervention characteristics

All included studies delivered conservative treatments for TMD, with or without control groups. Most of them used occlusal splint as the major intervention, with adjunct diet and lifestyle modification. A thin (ranged from 1.5–2.5mm), full-coverage upper hard acrylic splint, with even occlusal contact and a canine/anterior guidance occlusal scheme, to be worn only during sleep, was the most common protocol [19,20,40]. One study required patients to wear splints for upper or lower arches at all times, except during meals [43]. One study required patients to wear a splint during the day and/or night [22]. One study did not specify the design of splints [39]. Four studies used massage, a warm pack, and/or cryotherapy at masticatory muscles as interventions [22,37,38,42]. One study used anti-inflammatory medications as the only standardised intervention for TMD [41]. The duration of treatment ranged from 1–6 months (median: 2 months). One study did not specify the duration of treatment [41].

Among the eight RCTs included, only five studies fulfilled the definition of control group in this review. One RCT compared the occlusal splint to the placebo splint with the same wearing schedule [20]. The other four RCTs compared interventions for TMD to other non-standardised treatments, including counselling [19,22], self-care protocol [37], and aerobic exercise [42].

Outcome measures

Two studies [19,20] assessed the severity of both anxiety and depression to evaluate the outcome of interventions, while two [22,42] only assessed the severity of anxiety, and six [37,38,39,40,41,43] only assessed the severity of depression. The level of anxiety was assessed using the General Anxiety Disorder-7 [20,42], Hospital Anxiety and Depression Scale [19,22], and Beck Anxiety Inventory [22]. The level of depression was assessed using Beck’s Depression Inventory [38,43], the Symptom Checklist-90-Revised Instrument [37,39,40], the Patient Health Questionnaire-9 [20], and the Geriatric Depression Scale [41].

### 3.3. Quality of Studies

The quality of the 10 studies included is summarised and presented in Table 3. Three studies [20,22,42] were judged as “good” quality, six studies [19,37,38,39,40,43] were judged as “fair quality”, and one study [41] was judged as “poor quality”. Most of the studies that were judged as “poor” or “fair quality” were due to the lack of representativeness of the sample, small sample size, inadequate follow-up period, or lack of description to data lost.

### 3.4. Risk of Bias in Studies Included in the Meta-Analysis

**Figure 2:**
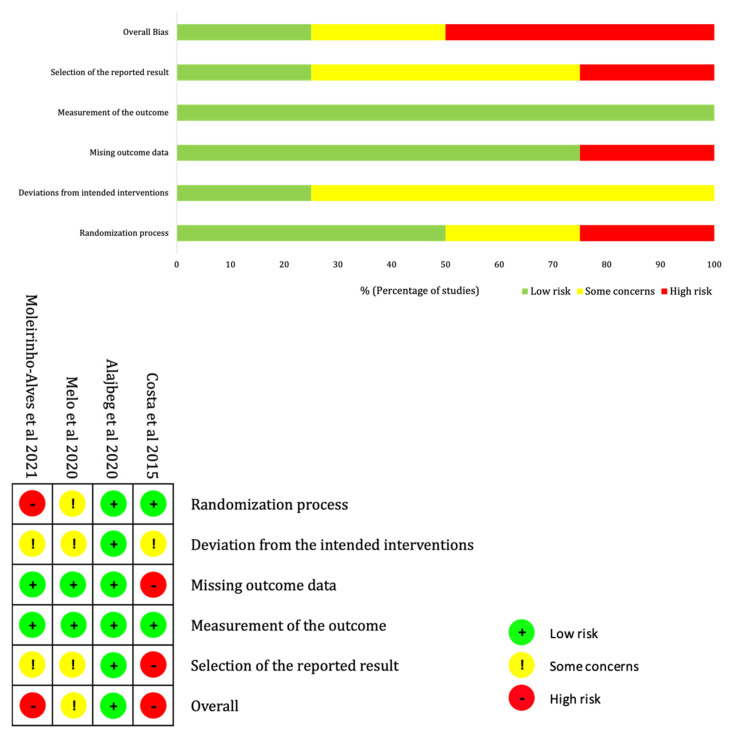
Summary and description of risk of bias assessment of the studies included in the meta-analysis using the Cochrane risk-of-bias tool for randomised trials (RoB 2) [19,20,22,42].

Only one RCT [20] was judged to have low risk of bias, one RCT [22] was judged to have some concern of bias, while the other two RCTs [19,42] were judged to have high risk of bias. The summary and description of the risk of bias assessment is presented in Figure 2. All studies had a low risk of bias in the measurement of the outcome, as they used common and standardised screening tools for the assessment of anxiety and/or depression with adequate description. The risk of bias in the randomization process was somewhat high because one RCT [22] did not report on adequate allocation concealment, and one RCT [42] did not allocate participants in a randomised manner, but according to participants’ preferences. The risk of bias in missing outcome data was high in one RCT [19] because of a high dropout rate of 32%, in which the number of dropped-out participants doubled in the control group compared to the test group, which was likely to induce bias in the result. The risk of bias in the selection of the reported result was generally of some concern or high because the numerical results reported in most of the studies were likely to be selected, such as the mean difference between the test and control groups were not always reported.

### 3.5. Narrative Analysis

Nine out of ten studies provided sufficient data regarding anxiety and/or depression to evaluate the overall effects of interventions over time, without controlling for the placebo effect for narrative analyses. Therefore, the results shall be interpreted with caution.

Anxiety

**Figure 3:**
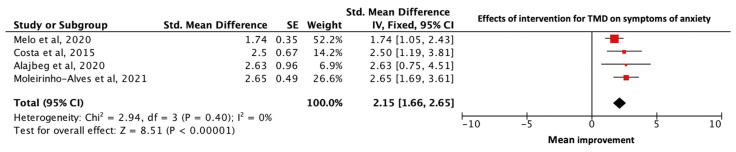
Forest plot of effects of intervention for TMD on symptoms of anxiety. Box size reflects study size. The diamond at the bottom reflects the overall pooled effect with a 95% confident interval. There was an overall significant mean improvement in symptoms of anxiety after interventions for TMD [19,20,22,42].

Four studies provided sufficient data regarding anxiety for the narrative analysis [19,20,22,42]. The combined data of the 129 participants who received interventions for TMD showed a statistically significant improvement in the symptoms of anxiety (SMD = 2.15; 95% CL 1.66 to 2.65; *p* < 0.00001). Very low and statistically insignificant heterogeneity was observed between studies (Heterogeneity: I^2^ = 0%; χ^2^ = 2.94; *p* = 0.40) (Figure 3).

Depression

Seven studies provided sufficient data regarding depression for the narrative analysis [19,20,38,39,40,41,43]. A random-effects model was employed to incorporate heterogeneity because a considerable number of studies were included [15]. The combined data of the 451 participants who received interventions for TMD showed a statistically significant improvement in symptoms of depression (SMD = 1.76; 95% CL 0.94 to 2.59; *p* < 0.0001). Strong and statistically significant heterogeneity was noted between studies (Heterogeneity: Tau^2^ = 0.97; I^2^ = 88%; χ^2^ = 51.84; *p* < 0.00001). Subgroup analyses regarding different interventions for TMD were performed.

**Figure 4:**
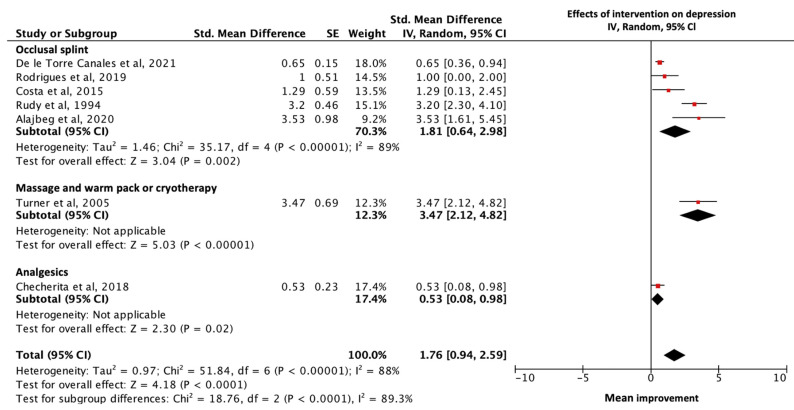
Forest plot of subgroup analysis of effects of intervention for TMD on symptoms of depression according to different interventions. Box size reflects study size. The diamond at the bottom reflects the overall pooled effect with a 95% confident interval. There was an overall significant mean improvement in symptoms of depression after interventions for TMD, as well as significant subgroup differences between different interventions [19,20,38,39,40,41,43].

Statistically significant improvement was observed in all three kinds of interventions. The test for subgroup differences suggested that a statically significant subgroup effect was evidenced (<0.0001). The treatment effect was greater for massage and warm pack or cryotherapy, followed by occlusal splint and analgesics (Massage and warm pack or cryotherapy: SMD = 3.47; 95% CL 2.12 to 4.82; *p* <0.00001; Occlusal splint: SMD = 1.81; 95% CL 0.64 to 2.98; *p* = 0.002; and Analgesics: SMD = 0.53; 95% CL 0.08 to 0.98; *p* = 0.02). However, there was unexplained heterogeneity between trials within the subgroup of occlusal splint (Tau^2^ = 1.46; I^2^ = 89%; χ^2^ = 35.17; *p* < 0.0001), which required further investigation (Figure 4).

Sensitivity analysis

In order to investigate factors contributing to the heterogeneity across studies, sensitivity analyses were performed by repeating the analyses according to the assessment tools used for depression. Mean differences for each tool were individually analysed. Statistically significant improvements in depression remained (BDI: MD = 3.28; 95% Cl 2.53 to 4; *p <* 0.00001; SCL-90R: MD = 0.68; 95% Cl 0.40 to 0.96; *p* < 0.00001), while no heterogeneity was observed within studies using the same assessment tool (BDI: I^2^ = 0%; χ^2^ = 0.11; *p* < 0.74; SCL-90R: I^2^ = 0%; χ^2^ = 0.43; *p* < 0.51) (Table 4).

### 3.6. Meta-Analysis

Four RCTs out of ten studies provided sufficient data regarding anxiety and/or depression for the meta-analysis to evaluate the overall effects of intervention over time, with the control of placebo effects. The summary of the results of the overall effects of intervention on anxiety and depression compared with the control group and the sensitivity analysis are presented in Table 5.

Anxiety

**Figure 5:**
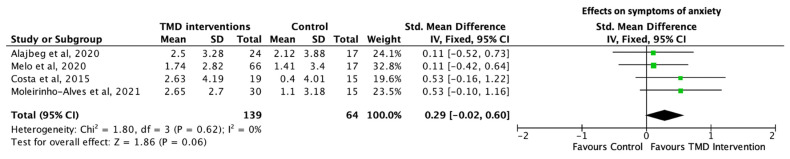
Forest plot of effects of intervention for TMD on symptoms of anxiety after controlling for placebo effect. Box size reflects study size. The diamond at the bottom reflects the overall pooled effect with a 95% confident interval. Positive SMD reflects effect on improving symptoms of anxiety favouring interventions for TMD over control. No statistically significant differences were observed between the two groups [19,20,22,42].

All four RCTs reported data regarding symptoms of anxiety, as evaluated using the GAD-7 questionnaire [20,42] or HADS [19,22]. The level of anxiety was assessed by two screening tools, HADS and BAI, in Melo’s RCT [22]. Data evaluated using HADS was extracted for this meta-analysis to minimise the heterogeneity between different screening tools. Analysis of these 4 studies (139 participants in the intervention arm and 64 participants in the control arm) showed no significant difference between the 2 groups (SMD = 0.29; 95% CL −0.02 to 0.60; *p* = 0.06) (Figure 5).

Depression

**Figure 6:**
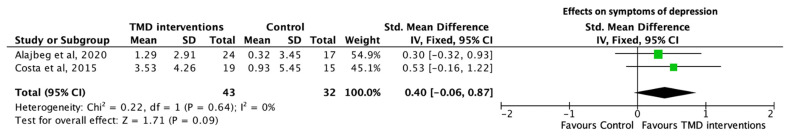
Forest plot of effects of interventions for TMD on symptoms of depression after controlling for placebo effect. Box size reflects study size. The diamond at the bottom reflects the overall pooled effect with a 95% confident interval. Positive SMD reflects effect on improving symptoms of depression favouring interventions for TMD over control. No statistically significant differences were observed between the two groups [19,20].

Only two RCTs reported sufficient data regarding symptoms of depression, as evaluated using the Patient Health Questionnaire-9 [20] or the HADS [19]. Analysis of these 2 studies (43 participants in the intervention arm and 32 participants in the control arm), showed no significant differences between the 2 groups (SMD = 0.40; 95% CL −0.06 to 0.87; *p* = 0.09) (Figure 6).

Sensitivity analysis

A sensitivity analysis was performed by repeating the meta-analysis regarding the effect of interventions on symptoms of anxiety after removing two studies [19,42] with a high risk of bias. The difference between the intervention and control groups remained insignificant (SMD = 0.11; 95% CL −0.3 to 0.51; *p* = 0.06). Sensitivity analyses were performed by repeating the meta-analyses, according to the assessment tools used for anxiety. The mean differences for each tool were individually analysed. However, there were still no significant differences between the intervention and control groups observed. Since all the studies delivered occlusal splints in their intervention arms, except Moleirinho-Alves et al. [42], which used massage and warm pack or cryotherapy as intervention, the analysis was repeated after removing its influence. Similarly, no significant differences between the intervention and control groups were observed (Table 5).

Quality of evidence

The level of certainty of the evidence was judged in the GRADE approach. Despite the low heterogeneity between studies in the analyses regarding both anxiety and depression, there were considerable risks of bias due to the generally small sample size in all studies, lack of blinding in both participants and clinicians in most studies, and high attrition rate in some studies. Therefore, it was deemed appropriate to downgrade the certainty of the evidence by two levels, from high to low, due to the imprecision of the results and the study limitations.

## 4. Discussion

### 4.1. Summary of the Findings

This systematic review and meta-analysis explored the best available evidence on the effectiveness of interventions for TMD on psychological outcome regarding symptoms of anxiety and depression in patients diagnosed with TMD. A total of 10 studies fulfilled the inclusion criteria and underwent qualitative analysis, while 9 studies provided sufficient data for the narrative analysis, and 4 RCTs for the meta-analysis. The results in all the studies generally suggested significant improvement in anxiety and depression after interventions for TMD, which is further demonstrated in our narrative analysis by an overall statistically significant reduction in the level of anxiety and depression. An obvious tendency of overall effects on improving symptoms in both depression and anxiety favouring interventions for TMD over control was observed in the meta-analyses; however, the effectiveness was found not statistically significant regarding a 95% confident interval. Furthermore, the subgroup analysis for the treatment effect on the improvement in depression regarding different interventions showed statically significant group differences, which in turn suggested that different interventions significantly modified the effect on the improvement in symptoms of depression. Heterogeneity was observed within subgroups, which suggested possible background factors that contributed to the varied results. In the sensitivity analysis, no heterogeneity was observed within studies using the same psychological assessment tools, suggesting that the use of various psychological assessment tools might be the reason for the heterogeneity.

### 4.2. Role of Interventions for TMD in Improving Anxiety and Depression

The statistically significant effect observed in the narrative analysis suggests a beneficial effect of interventions for TMD on reducing levels of depression and anxiety, regardless of the types of interventions given. The mechanism of this beneficial effect was suggested to be associated with the relationship between pain and TMD. Previous studies have indicated the mutual interaction between pain and psychological distresses [14,44] Successful therapeutic treatments in patients with TMD are suggested to have a positive effect in improving symptoms of anxiety and depression by pain management strategies [45].

### 4.3. Statistical Significance Not Found in Treatment Effect When Compared to Control Group

The overall treatment effect tended to favour interventions for TMD over the control in improving symptoms of depression and anxiety. However, it was not found to be statistically significant. This finding was likely because only a limited number of eligible studies were included in this meta-analysis [46]. Furthermore, most studies [19,22] provided treatments such as counselling to patients in the control group; only one study [20] used placebo splint in the control setup. These non-standardised interventions have likely resulted in a positive effect on the psychological outcomes, which have in turn weakened the effects of the standardised interventions shown in the statistics.

### 4.4. Implication for Clinical Practice

This review suggested a supportive role of interventions for TMD in improving anxiety and depression. It is demonstrated in the Turner et al. [38] and Costa et al. [19] studies that the combination of treatments for TMD and psychological interventions, such as cognitive-behavioural therapy and counselling, resulted in the best outcome. They believed the involvement of a psychological approach allowed relaxation and better pain-coping strategies which worked hand-in-hand with the standardised interventions in the management of TMD. Previous studies also supported the implication of psychosocial interventions for chronic orofacial pain [47]. On the other hand, it is also important for psychologists to be aware of any signs of TMD in their patients. A timely referral to oral surgeons might help in the management of psychological distress of their patients. A multidisciplinary approach is suggested to best manage this multifactorial illness.

### 4.5. Implication for Future Research

Future RCTs should ensure the high quality of the methodology and reporting, including larger sample sizes, allocation concealment, control groups with no treatments or placebo treatments, and intention-to-treat analyses. Meta-analyses could be repeated when there are more eligible studies available to improve generalization and obtain an accurate overall treatment effect. Future RCTs could be conducted to compare the effectiveness between standardised interventions for TMD; psychological interventions; and combinations of both and no treatments, on both pain control and psychological outcomes. This requires contributions of expertise from both oral surgery and psychology.

### 4.6. Strengths and Limitations of This Review

There were several limitations in this review. First, only a small number of studies could be included in this meta-analysis. The pooled sample size was relatively small to identify significant relationships within the dataset.

Secondly, high heterogeneity existed in the various assessment scales of anxiety and depression applied in different studies. Multiple cut-off points were used among studies that used the same assessment tools. The duration of intervention varied, and measurements of outcome parameters were obtained at different time-points across studies. These have made direct comparison of the study outcomes difficult. The summary statistics required for meta-analysis were unavailable in most studies, and much statistical estimation was performed, which might induce inaccuracy in the analysis.

Furthermore, the low methodological quality of the available RCTs might also include bias. Since all the assessment tools of anxiety and depression relied on questionnaires completed by patients, blinding of outcome measurements became impossible. Some studies did not conduct intention-to-treat, but rather per-protocol analyses when there were missing data.

In addition, the studies that fulfilled the inclusion criteria, and thus were included in this systematic review, consisted only of a limited array of the currently available treatment options, such as occlusal splint and anti-inflammatory medications. Studies pertaining to other common interventions for TMD, such as intra-articular injection and arthrocentesis, which also fulfil the inclusion criteria of this systematic review, were not found. It is, therefore, not possible to relate the findings of the current systematic review and meta-analysis to those other common interventions for TMD.

Lastly, the patients included in the studies were mostly psychological healthy individuals with symptoms, but not diagnosed with anxiety and depression. The difference before and after interventions might, therefore, be too small to be reflected in the statistics.

Nevertheless, to the best of our knowledge, this is the first systematic review and meta-analysis to evaluate the effectiveness of interventions for TMD in reducing psychological distress. A comprehensive search of available literature was conducted, with an established review methodology applied, to minimise possible bias. Although only a handful of studies could be included in the meta-analysis, we attempted to summarise the best available evidence and identify the current research gap in this topic. This systematic review and meta-analysis serves as an exploratory review, providing a plausible estimate that could be tested in the future in subsequent reviews of the role of interventions for TMD in correcting psychological stress.

## 5. Conclusions

This systematic review and meta-analysis have suggested the interventions for TMD may be beneficial in improving symptoms of depression and anxiety, based on the current available evidence. However, the effect is statistically uncertain and warrants future studies to enable the best synthesis of the evidence. Multidisciplinary management, with the input of both the surgeons and the psychologists, is recommended in treating patients presented with TMD and symptoms of psychological distress.

## Figures and Tables

**Table 1:** Databases searched, search terms used, and number of articles found per database.

Database Searched	Search Terms	Articles Retrieved
Pubmed	(“Temporomandibular Joint Disorders” or “Temporomandibular joint disorder” or “TMJ Disorders” or “TMJ Disorder” or “Temporomandibular Disorders” or “Temporomandibular Disorder” or “Temporomandibular Joint Diseases” or “Temporomandibular Joint Disease” or “TMJ Diseases” or “TMJ Disease” or “Temporomandibular joint dysfunction syndrome” or “Temporomandibular joint pain” or “Temporomandibular pain” or “TMD” or “Craniomandibular Disorders” or “Craniomandibular Disorder” or “Orofacial Pain” or “Craniofacial pain”) AND (“Depression” or “depressive disorders” or “depression symptoms” or “anxiety” or “mood disorders” or “psychological distress”)	1285
Web of Science	(“Temporomandibular Joint Disorders” or “Temporomandibular joint disorder” or “TMJ Disorders” or “TMJ Disorder” or “Temporomandibular Disorders” or “Temporomandibular Disorder” or “Temporomandibular Joint Diseases” or “Temporomandibular Joint Disease” or “TMJ Diseases” or “TMJ Disease” or “Temporomandibular joint dysfunction syndrome” or “Temporomandibular joint pain” or “Temporomandibular pain” or “TMD” or “Craniomandibular Disorders” or “Craniomandibular Disorder” or “Orofacial Pain” or “Craniofacial pain”) AND (“Depression” or “depressive disorders” or “depression symptoms” or “anxiety” or “mood disorders” or “psychological distress”)	1387
Medline	(Temporomandibular Joint Disorders or Temporomandibular joint disorder or TMJ Disorders or TMJ Disorder or Temporomandibular Disorders or Temporomandibular Disorder or Temporomandibular Joint Diseases or Temporomandibular Joint Disease or TMJ Diseases or TMJ Disease or Temporomandibular joint dysfunction syndrome or Temporomandibular joint pain or Temporomandibular pain or TMD or Craniomandibular Disorders or Craniomandibular Disorder or Orofacial Pain or Craniofacial pain) and (Depression or depressive disorders or depression symptoms or anxiety or mood disorders or psychological distress)	1027
Cochrane	(“Temporomandibular Joint Disorders” or “Temporomandibular joint disorder” or “TMJ Disorders” or “TMJ Disorder” or “Temporomandibular Disorders” or “Temporomandibular Disorder” or “Temporomandibular Joint Diseases” or “Temporomandibular Joint Disease” or “TMJ Diseases” or “TMJ Disease” or “Temporomandibular joint dysfunction syndrome” or “Temporomandibular joint pain” or “Temporomandibular pain” or “TMD” or “Craniomandibular Disorders” or “Craniomandibular Disorder” or “Orofacial Pain” or “Craniofacial pain”) AND (“Depression” or “depressive disorders” or “depression symptoms” or “anxiety” or “mood disorders” or “psychological distress”)	237
Scopus	(“Temporomandibular Joint Disorders” OR “Temporomandibular joint disorder” OR “TMJ Disorders” OR “TMJ Disorder” OR “Temporomandibular Disorders” OR “Temporomandibular Disorder” OR “Temporomandibular Joint Diseases” OR “Temporomandibular Joint Disease” OR “TMJ Diseases” OR “TMJ Disease” OR “Temporomandibular joint dysfunction syndrome” OR “Temporomandibular joint pain” OR “Temporomandibular pain” OR “TMD” OR “Craniomandibular Disorders” OR “Craniomandibular Disorder” OR “Orofacial Pain” OR “Craniofacial pain”) AND (“depression” OR “depressive disorders” OR “depression symptoms” OR “anxiety” OR “mood disorders” OR “psychological distress”)	1656

**Table 2:** Summary of characteristics of included studies.

Study	Country	Characteristics of Subjects - Number of Subjects at - Baseline - Age at Baseline - Female to Male Ratio	Intervention Groups - Number of Subjects in each Group - Description of Interventions - Description of Control (if any) - Duration of Treatment		Duration of Follow-Up	Diagnostics Tool of TMD	Outcome Measures of Anxiety or/and Depression	Results
			Experimental	Control				
Brandão et al., 2022 [37]	Brazil	- 23 Adults diagnosed with TMD - Recruited from the Center for Health and Functional Studies at the Health Sciences Institute of the Federal University of Bahia - Mean age 35.9 ± 10.5 **^†^** - 84.2% Female	- 12 Subjects - Circular massage; at masseter muscle; for 5-min - Pain relief exercise; 30-min session - By researcher; twice weekly; for 4 weeks	- 11 Subjects - Self-care protocol: avoid opening mouth widely, hard food and oral parafunction - Reassess after 30 days	- 1 month	RDC/TMD	Depression: RDC/TMD Axis II	- Improvement in depression demonstrated in the intervention groups with a considerable effect size - Significance not reported
De la Torre Canales et al., 2021 [40]	Brazil	- 20 Female diagnosed with myofascial pain (There were a total of 100 subjects in the paper, evenly distributed in the occlusal appliance group, saline injection group and three groups of botulinum toxin injection in different preparation. However, botulinum injection is defined as non-standardised intervention in this review, only the occlusal appliance group is included in the analysis.) - Recruited from the TMD clinic of Piracicaba Dental School, University of Campinas, São Paulo, Brazil - Mean age 36.8 ± 5.6 - 100% Female	- 20 Subjects - Occlusal appliance - Full coverage; flat; heat-cured acrylic; for upper arch only; canine and anterior guidance occlusal scheme - To wear only during sleep - 6 months	- No control groups	- 6 months	RDC/TMD	Depression: SCL-90R	- Significant improvement in depression demonstrated after treatment
Moleirinho-Alves et al., 2021 [42]	Portugal	- 52 Adults diagnosed with myalgia - Recruited from the Egas Moniz University Clinic and the Egas Moniz Dental Clinic - Mean age 25.9 ± 4.5 **†** - 86.7% Female	- Total 22 Subjects - 15 Subjects in the therapeutic group; physiotherapy consisted of massage of masticatory muscles, isotonic strengthening and coordination exercises by physiotherapist; 30-min session; weekly; for 8 weeks - 17 Subjects in the therapeutic and aerobic exercise group; weekly 30-min physiotherapy and two weekly 30-min aerobic programme; for 8 weeks	- 20 Subjects - Aerobic exercise group - Two weekly cycle ergometer training sessions, for 8 weeks	- 2 months	DC/TMD	Anxiety: GAD-7	- All groups showed small improvement (less than 4 points) in anxiety after treatments Minimum clinically important difference defined as reduction of four points in the total score in GAD-7. - Statistical significance not reported - However, no between-group differences
Alajbeg et al., 2020 [20]	Croatia	- 34 Patients diagnosed with TMD - Recruited from patients seeking treatment for orofacial pain at the School of Dental Medicine, - University of Zagreb - Mean age 36.1 ± 11.95 years - 100% Female	- 19 Subjects - Stabilization splint - Full coverage; hard acrylic; for upper arch only; centric relation; 1.5 mm thickness; smooth and flat surface with canine guidance occlusal scheme - To wear it only at night during sleep - 6 months	- 15 Subjects - Placebo splint - A thin transparent foil of 0.5 mm thickness - To wear it only at night during sleep - 6 months	- 3 months - 6 months	DC/TMD	Anxiety: GAD-7Depression: PHQ-9	- The intervention group presented significantly greater reduction in both anxiety and depression at post-treatment relative to baseline, compared to placebo
Melo et al., 2020 [22]	Brazil	- 112 Adults diagnosed with TMD - Recruited by the Department of dentistry, the Federal - University of Rio Grande do Norte - Mean age 28 ± 9.34 - 82.1% Female	- Total 84 Subjects - 28 Subjects in the occlusal splint group; full coverage; hard acrylic; for upper arch only; even interocclusal contact in centric relation position; canine guidance occlusal scheme; wear at night and/or daytime; adjustments after 15 days - 28 Subjects in the occlusal splint with counselling group; - Reinforcement after 15 days - 28 Subjects in the manual therapy group; heat and cryotherapy, therapeutic exercises; performed by a trained researcher, 40-min session, twice per week, for 4 weeks	- 28 Subjects - Counselling only - Written booklet with dietary guidelines, physical exercises, lifestyle modification - Individualized investigation of possible aetiology and orientated guidelines - Reinforcement after 15 days	- 1 month	RDC/TMD	Anxiety: HADS, BAI	- All groups presented significant improvement in anxiety after 1 month of treatment - However, no between-group differences
Rodrigues et al., 2019 [39]	Brazil	- 20 Adults diagnosed with myofascial pain (There were a total of 40 subjects in the paper, evenly distributed in the laser auriculotherapy (LA) and the occlusal splint (OS) group. However, LA is defined as non-standardised intervention in this review, the 20 subjects in the LA group is excluded from the analysis.) - Recruited from a pool of primary school teachers in the city Campina da Lagoa, Paraná, Brazil - Mean age 43.63 - 100% Female	- 20 Subjects - Occlusal splint - Design of splint not specified - For 8 h overnight - Occlusal adjustment after 2 and 7 days - 8 weeks	- No control groups	- 2 months	RDC/TMD	Depression: SCL-90	- Significant improvement in depression demonstrated at post-treatment
Checherita et al., 2018 [41]	Romania	- 107 Elderly (aged 60–79 years) diagnosed with TMD - Recruited from two private dental offices and the Mihail Kogalniceanu Clinical Education Base, of Iasi - Mean age 68.72 ± 8.37 years - 69.1% Female	- 107 Subjects - Anti-inflammatory medication - Ibuprofen 800–1200 mg/day, for 7–14 days - Used the lowest effective dose and shortest treatment duration	- No control groups	- Not specified	RDC/TMD	Depression: GDS	- Depressive manifestation improved at post-treatment, demonstrated as increased proportion of elderly with no depressive symptoms after treatment - Significance not reported
Costa et al., 2015 [19]	Brazil	- 60 Adults diagnosed with masticatory myofascial pain - Recruited from local community through advertisements - Mean age 31.85 ± 7.81 **†** - 90% Female	- 30 Subjects - Occlusal splint and counselling - Full coverage; hard acrylic; for upper arch only; 2–2.5 mm thickness; smooth and flat surface with anterior guidance occlusal scheme - To wear it only at night during sleep - 5 months	- 30 Subjects - Counselling only - Verbal and written information about TMD aetiology and prognostics, diet modification, lifestyle modification, relaxation of jaw, warm pack, self-massage - 5 months	- 2 months - 5 months	RDC/TMD	Anxiety: HADS, Depression: HADS	- Only the intervention group demonstrated significant reduction in anxiety and depression - However, no between-group differences
Turner et al., 2006 [38]	USA	- 158 Adults diagnosed with TMD - Recruited from the University of Washington Orofacial Pain Clinic - Mean age 37.0 ± 11.4 - 86% Female	- Total 158 subjects - All Subjects received conservative interventions for TMD: jaw exercise, warm and/or cold pack, diet modification - Some were prescribed medications and occlusal splints - 79 Subjects in in the pain management training (PMT) group; a brief cognitive-behavioural therapy conducted by a psychologist - 79 subjects in the self-care management (SCM); an education/attention control condition conducted by a bachelor’s level educator trained by a psychologist - Biweekly; for 8 weeks	- No control groups	- 2 months - 6 months - 12 months	RDC/TMD	Depression: BD [14]	- Improvement in depression demonstrated in both intervention groups at all post-treatment assessments - Significance not reported - The PMT group presented a significantly higher improvement than the SCM group at 12 months
Rudy et al., 1995 [43]	USA	- 150 Adults diagnosed with TMD - Recruited from an outpatient TMD clinic at the University of Pittsburgh Medical Center - Mean age 32.4 ± 8.4 - 89% Female	- 150 Subjects - Interocclusal appliance and biofeedback/stress management treatment - Full coverage; heat-cured; flat; upper or lower arch; 1–2 mm thickness; even contact in centric relationship; canine guidance occlusal scheme - To wear all times for the first 4 weeks, except during meals and oral hygiene - Biofeedback-assisted relation procedures and stress management treatment conducted by psychologist; 75-min session; weekly; for 6 weeks	- No control groups	- 6 weeks (not reported) - 6 months	RDC/TMD	Depression: BDI	- Significant improvement in depression demonstrated at 6 months follow-up

**^†^** Combined mean and SD of age derived from Cochrane’s Formula. GAD-7, General Anxiety Disorder-7; PHQ-9, Patient Health Questionnaire-9; GDS, Geriatric Depression Scale; HADS, Hospital Anxiety and Depression Scale; BAI, Beck Anxiety Inventory; SCL-90, Symptoms Checklist-90; BDI, Beck Depression Inventory.

**Table 3:** Quality assessment of studies using the modified Newcastle–Ottawa Scale.

Study	Selection	Comparability	Outcome	Total Score
Alajbeg et al., 2020 [20]	***	*	***	7
Melo et al., 2020 [22]	***	*	**	6
Moleirinho-Alves et al., 2021 [42]	***	*	**	6
Costa et al., 2015 [19]	***	*	*	5
De la Torre Canales et al., 2021 [40]	**	*	**	5
Rudy et al., 1995 [43]	**		***	5
Turner et al., 2006 [38]	**	*	**	5
Brandão et al., 2022 [37]	*	*	**	4
Rodrigues et al., 2019 [39]	**	*	*	4
Checherita et al., 2018 [41]			**	2

Total score of 6–7: good quality; 3–5: fair quality; and 0–2: poor quality. *, **, *** Represents the score awarded in each section.

**Table 4:** Summary of the narrative analysis of effects of interventions for TMD on severity of anxiety and depression and sensitivity analyses.

	Number of Studies	Included Studies	Number of Participants (Intervention)	SMD (95% Cl)	*p* Value	Heterogeneity I2; χ2; P
**Intervention effects**						
Anxiety	4	[19,20,22,42]	139	2.15 (1.66–2.65)	<0.00001	0%; 2.94; 0.40
Depression	7	[19,20,38,39,40,41,43]	451	1.76 (0.94–2.59)	<0.0001	88%; 51.84; <0.00001
**Sensitivity analysis (Depression)**						
Assessed by BDI	2	[38,43]	270	3.28 (2.53, 4.03) *	<0.00001	0%; 0.11; 0.74
Assessed by SCL-90R	2	[39,40]	31	0.68 (0.40, 0.96) *	<0.00001	0%; 0.43; 0.51

* Mean differences instead of SMD were estimated because the same assessment tools were used in the studies included. SMD, Standard mean difference; BDI, Beck’s Depression Index; SCL-90R, Screening Checklist—90 Revised.

**Table 5:** Summary of effects of interventions for TMD on severity of anxiety and depression and sensitivity analysis.

	Number of Studies	Included Studies	Number of Participants (Intervention)	Number of Participants (Control)	SMD (95% Cl)	*p* Value	Heterogeneity I2; χ2; P
**Intervention effects**							
Anxiety	4	[19,20,22,42]	139	64	0.29 (0.02–0.6)	0.06	0%; 1.80; 0.62
Depression	2	[19,20]	43	32	0.40 (−0.06–0.87)	0.09	0%; 0.22; 0.64
**Sensitivity analysis (Anxiety)**							
Higher-quality studies	2	[20,22]	90	34	0.11 (−0.3–0.51)	0.06	0%; 0.00; 0.99
Occlusal splints	3	[19,20,22]	109	49	0.22 (−0.13–0.57)	0.22	0%; 1.06; 0.59
Assessed by GAD−7	2	[20,42]	54	32	1.07 (−0.37, 2.52) *	0.15	0%; 0.61; 0.44
Assessed by HADS	2	[19,22]	85	32	0.87 (−0.61, 2.36) *	0.25	23%; 1.29; 0.26

* Mean differences instead of SMD were estimated because the same assessment tools were used in the studies included. SMD, Standard mean difference; GAD-7, General Anxiety Disorder–7; HADS, Hospital Anxiety and Depression Scale.

## Data Availability

Data is available in the manuscript.

## Appendix A

**Modified Newcastle–Ottawa Quality Assessment Scale**

**For clinical trials**

Note: A study can be awarded a maximum of one star for each numbered item. A maximum of seven stars is available in total, including three stars in “Selection”, one star in “Comparability”, and three stars in “Outcome”.

**Selection**

1. Representativeness of the sample.
  (a) Truly representative of the average in patients with TMD (Random sampling/population based sampling)*.
  (b) Somewhat representative of the average in patients with TMD (non-random sampling)*.
  (c) Selected group of patients, e.g., volunteer, students, hospital staffs, restricted by gender, etc.
  (d) No description of the sampling strategy.
2. Sample size.
  (a) Justified and satisfactory, that is, to have a clear description of algorithm, the required sample size is derived*.
  (b) Not justified.
3. Ascertainment of intervention.
  (a) Protocol of intervention is clearly described. Attempts are made to ensure the intervention is accurately carried out*.
  (b) Protocol of intervention is somewhat described, but no confirmation of whether the intervention is accurately carried out.
  (c) No description of intervention or no attempt to ascertain information on how accurate the intervention is carried out.

**Comparability**

(a) Presence of a control or comparable group, e.g., placebo, no treatment, or other non-standardised interventions for TMD*.

**Outcome**

1. Assessment of outcome
  (a) Independent blind assessment*
  (b) Record linkage*
  (c) Self-report*
  (d) No description
2. Was follow-up long enough for outcomes to occur
  (a) Yes (length of intervention and follow-up was at least 6 months)*
  (b) No
3. Adequacy of follow-up
  (a) Complete follow up—all subjects accounted for*
  (b) Number of subjects lost to follow-up is more and unlikely to introduce bias (<20%), or description of those lost is clearly reported*
  (c) >20% of subjects lost to follow-up and no description of those lost
